# Genetic Variation of *Bordetella pertussis* in Austria

**DOI:** 10.1371/journal.pone.0132623

**Published:** 2015-07-16

**Authors:** Birgit Wagner, Helen Melzer, Georg Freymüller, Sabine Stumvoll, Pamela Rendi-Wagner, Maria Paulke-Korinek, Andreas Repa, Frits R. Mooi, Herwig Kollaritsch, Helmut Mittermayer, Harald H. Kessler, Gerold Stanek, Ralf Steinborn, Michael Duchêne, Ursula Wiedermann

**Affiliations:** 1 Institute of Specific Prophylaxis and Tropical Medicine, Center for Pathophysiology, Infectiology and Immunology, Medical University of Vienna, Vienna, Austria; 2 Astellas Pharma, Vienna, Austria; 3 Genomics Core Facility, VetCore, University of Veterinary Medicine, Vienna, Austria; 4 Department of Hygiene, Microbiology and Tropical Medicine, Elisabethinen Hospital, Linz, Austria; 5 analyse BioLab, Linz, Austria; 6 Federal Ministry of Health, Vienna, Austria; 7 Department of Pediatrics and Adolescent Medicine, Medical University of Vienna, Vienna, Austria; 8 Centre for Infectious Diseases Control, National Institute of Public Health and the Environment, Bilthoven, The Netherlands; 9 Department of Hygiene, Microbiology and Environmental Medicine, Medical University Graz, Graz, Austria; 10 Institute for Hygiene and Applied Immunology, Center for Pathophysiology, Infectiology and Immunology, Medical University of Vienna, Vienna, Austria; St. Petersburg Pasteur Institute, RUSSIAN FEDERATION

## Abstract

In Austria, vaccination coverage against *Bordetella pertussis* infections during infancy is estimated at around 90%. Within the last years, however, the number of pertussis cases has increased steadily, not only in children but also in adolescents and adults, indicating both insufficient herd immunity and vaccine coverage. Waning immunity in the host and/or adaptation of the bacterium to the immunised hosts could contribute to the observed re-emergence of pertussis. In this study we therefore addressed the genetic variability in *B*. *pertussis* strains from several Austrian cities. Between the years 2002 and 2008, 110 samples were collected from Vienna (n = 32), Linz (n = 63) and Graz (n = 15) by nasopharyngeal swabs. DNA was extracted from the swabs, and bacterial sequence polymorphisms were examined by MLVA (multiple-locus variable number of tandem repeat analysis) (n = 77), by PCR amplification and conventional Sanger sequencing of the polymorphic regions of the *prn* (pertactin) gene (n = 110), and by amplification refractory mutation system quantitative PCR (ARMS-qPCR) (n = 110) to directly address polymorphisms in the genes encoding two pertussis toxin subunits (*ptxA* and *ptxB*), a fimbrial adhesin (*fimD*), tracheal colonisation factor (*tcfA*), and the virulence sensor protein (*bvgS*). Finally, the *ptxP* promoter region was screened by ARMS-qPCR for the presence of the *ptxP3* allele, which has been associated with elevated production of pertussis toxin. The MLVA analysis revealed the highest level of polymorphisms with an absence of MLVA Type 29, which is found outside Austria. Only Prn subtypes Prn1/7, Prn2 and Prn3 were found with a predominance of the non-vaccine type Prn2. The analysis of the *ptxA*, *ptxB*, *fimD*, *tcfA* and *bvgS* polymorphisms showed a genotype mixed between the vaccine strain Tohama I and a clinical isolate from 2006 (L517). The major part of the samples (93%) displayed the *ptxP3* allele. The consequences for the vaccination strategy are discussed.

## Introduction

*Bordetella pertussis*, the causative agent of whooping cough, is still responsible for significant morbidity and mortality in many countries of the world. Mainly in resource-poor regions, every year 195,000 children, mostly unimmunised, are estimated to die from pertussis [1].

To control pertussis, whole cell vaccines (WCVs) were introduced in the 1940s and have been replaced by the less reactogenic acellular vaccines (ACVs) in the 1980s. ACVs contain the detoxified pertussis toxin (Ptx), filamentous hemagglutinin (FHA), pertactin (Prn) and fimbriae (Fim) in various combinations. A recent review from the Cochrane Acute Respiratory Infections Group [2] summed up six efficacy trials with a total of 46,283 participants. Multi-component ACVs (n ≥ 3 components) had an efficacy between 84% and 85% in preventing typical whooping cough. In contrast, the examined one- or two-component ACVs prevented only between 59% and 75% of typical whooping cough, however, a new Danish monocomponent ACV, containing only Ptx toxoid, was recently shown to achieve excellent 93% efficacy against severe pertussis requiring hospitalisation [3]. The Cochrane Group also assessed 52 safety trials with a total of 136,541 participants to show that indeed the ACVs lead to significantly fewer local and systemic adverse reactions than the WCVs [2].

In Austria, the pertussis vaccination coverage is estimated at around 90% for infant vaccination. A hexavalent vaccine is given including the pertussis ACV combined with vaccines against diptheria, tetanus, polio, hepatitis B and *Haemophilus influenzae* type B. For booster vaccination, a tetravalent vaccine without the two last components is given. In many affluent countries like Austria, despite high pertussis vaccination coverage, increasing incidence rates have been observed [4–8]. On the average, the most affected group was the 5–14-year-olds with a confirmed case rate slightly higher than 11 per 100,000 population [9]. In Austria the all-time low in the number of pertussis cases was in 1995 with only 91 reported cases (1.14 per 100,000 population) and since then these numbers have been increasing continuously up to 425 confirmed cases (5.06 per 100,000 population) in 2012 [9]. Although the disease in older children is milder than in the infants, there are concerns that small children may be infected before their first vaccination. In addition, severe pertussis cases have been increasingly observed in the elderly population [7]. Consequently, it is important to understand and to try to stop the re-emergence of pertussis cases.

Several causes are conceivable for the rise in incidence. The rise in awareness and improvement of diagnostics could play a certain role, but probably this will be limited. More likely, some cases of pertussis in older children and adults may not be diagnosed correctly, as the symptoms may be atypical or mild, and since diagnostic procedures such as culture may have a low sensitivity [10]. Another explanation is the waning protection [11,12] against *B*. *pertussis* accompanied by waning antibody levels [13], which have been observed in children immunised with ACVs.

A third explanation for the increasing pertussis prevalence would be that under the pressure of vaccination, altered *B*. *pertussis* strains might emerge which would be less affected by the immune reaction of vaccinated individuals. An early study [14] analysed a large Dutch pertussis strain collection for their polymorphisms in the *prn* and *ptxA* genes. While the older strains displayed the same gene variants as the vaccine strains, the newer strains collected between 1990 and 1996 were predominantly non-vaccine types. As there were large differences in the vaccination schedule and the composition of the vaccines at the time, a surveillance program entitled “European Research Programme for Improved Pertussis Strain Characterization and Surveillance (EUpertstrain)”was founded to monitor differences in the incidence of pertussis and in the population structure of *B*. *pertussis* in Europe [15,16].

Few years later, a new method, multiple-locus variable-number tandem repeat analysis (MLVA) was introduced [17] which examines highly polymorphic regions containing variable numbers of tandem repeats (VNTRs). This method also showed that pre-1990 pertussis strains were significantly more diverse than the strains isolated post-1990, and that some strains expanded clonally during epidemic periods. MLVA (VNTR-typing) and sequence analysis of the *prn*, *ptxA*, *ptxC*, and *tcfA* genes in 102 strains, isolated in the period from 1998 to 2001 in Finland, Sweden, Germany, the Netherlands and France, revealed a relationship between VNTR subtypes and geographic origin, but no relationship to vaccination programs was observed [18].

In 2003, the *B*. *pertussis* genome was published and compared with the genomes of the less pathogenic *Bordetella parapertussis* and *Bordetella bronchiseptica* [19]. In the search of further polymorphisms between *B*. *pertussis* strains, the sequence of the older genome strain Tohama I was compared with the strain L517 isolated in Australia in 2006 [20]. In total 70 single nucleotide polymorphisms (SNPs) as well as five indels of 1–3 basepairs were detected. Five non-synonymous base changes in virulence-associated genes were among the SNPs.

A major review [21] illustrates the low level of sequence variation in *B*. *pertussis* in general, nevertheless, this review also cites 42 studies which describe polymorphisms and temporal shifts in *B*. *pertussis* alleles in various countries. The most variable cell envelope component is pertactin (Prn). The variations of Prn are confined to two regions, however. Region 1 contains repeats comprising the pentapeptide elements GGAVP, GGFGP and GGGVP in eleven different combinations, and in region 2, four more single amino acid polymorphisms are found. Altogether there are 13 variants Prn1 to Prn13. An important variant of a non-protein coding sequence is the *ptxP3* allele which comprises a change in the pertussis toxin promoter region which is associated with a higher expression of Ptx [22] and a number of other virulence factors, and with increased virulence. This strain was shown to expand significantly in the Netherlands so it could contribute to the pertussis resurgence [23].

The comparative genomic sequencing of two strains had revealed only one SNP per 20 kb [20], and in total, polymorphisms were found in only 20 cell envelope- and virulence-associated proteins, so *B*. *pertussis* can be called a monomorphic pathogen [21]. Nevertheless, when comparing different strains, but even after passaging the strain Tohama I in the laboratory, large chromosomal rearrangements can be observed [24]. These can be caused by recombination of insertion sequence elements (ISEs). As an example, strain Tohama I contains 238 copies of IS*481* [19], corresponding to about 6% of its total genome. In strains from Finland [25] and the Netherlands [26] isolated during the past decades, progressive gene loss was observed, with lost loci flanked by IS*481* elements, and the authors suggest that this could be a strategy of *B*. *pertussis* to adapt to highly immunised populations. Moreover, strains from Belgium and the Netherlands were discovered, in which the whole *tcfA* gene was deleted, presumably by recombination also involving repeated sequences flanking the gene [27]. More recently, strains have emerged which do not produce Prn, a component of most vaccines [28,29].

In the present study, we applied several methods to investigate gene polymorphisms in Austrian *B*. *pertussis* isolates. Genotyping was done by means of MLVA analysis [17], Sanger amplicon sequencing of the *prn* gene polymorphic region 1 [21], and ARMS-qPCR (amplification refractory mutation system quantitative PCR) [30] for testing of five SNPs in virulence-associated proteins [20] and for identification of the *ptxP3* allele associated with increased expression of pertussis toxin [23].

## Results

*B*. *pertussis* samples (n = 110) from patients aged from 1 month to 47 years were collected from 2002 to 2008 in three Austrian cities (Vienna, Linz, and Graz). The first part, collected from 2002 to 2006 (n = 77) was characterised by MLVA typing [17], and all samples (n = 110) were examined by sequencing of the *prn* gene polymorphic region-1 [21], and ARMS-qPCR [30] of target SNPs in the genes coding for pertussis toxin (*ptxA* and *ptxB*), fimbrial adhesin (*fimD*), tracheal colonisation factor (*tcfA*), the virulence sensor protein (*bvgS*) [20], and the pertussis toxin promoter region *ptxP* [22]. The detailed results can be found in S1 Table.

### MLVA typing shows significant genotypic diversity of *B*. *pertussis* strains in Austria

For the initial study on MLVA typing, 77 *B*. *pertussis* samples of infected patients with ages between one month and 47 years (median 5.6 years of age) were collected from 2002 to 2006 in three Austrian cities. Typing was successful for 34 of these samples. Seven different MLVA types were identified (Table 1). The most common MLVA type was Type 27 with a frequency of 67% (23/34) corresponding to the data from other European countries [18]. MLVA type 29, which had been prevalent in other parts of Europe before 2000, was not found in any of the Austrian samples. Instead, we detected various other MLVA types, *i*.*e*. Types 18, 36, 38, 63, 128 and 129 with frequencies between 3% and 12%. The genotypic diversity (GD) in the Austrian *B*. *pertussis* population based on MLVA types was 0.56, similar to the GDs of the Swedish (GD = 0.56), Dutch (GD = 0.52), French (GD = 0.51) and German (GD = 0.62) samples but less than the GD of the Finnish (GD = 0.80) samples [18].

**Table 1:** Variation of MLVA types in Austrian *B*. *pertussis* samples (n = 34) collected from 2002–2006. Frequencies are given as numbers and as percentage of successfully typed samples (in brackets).

MLVA type	Number and percentage
18	1 (3%)
27	23 (67%)
36	2 (6%)
38	4 (12%)
63	2 (6%)
128	1 (3%)
129	1 (3%)

### Analysis of prn polymorphisms in the Austrian samples shows three variants

In this second part of the study, including the first 77 samples plus additional 33 samples from Austrian patients (n = 110), the major polymorphic region 1 of the *prn* gene was PCR-amplified and 77 of the 110 *B*. *pertussis* samples could be sequenced successfully. Out of the 13 known Prn variants [21], only three, Prn1/7, Prn2 and Prn3 were found in Austria (Table 2). The Prn2 variant predominated in strains isolated in all three Austrian cities with frequencies of 64% (Graz), 72% (Vienna) and 76% (Linz). Overall, 56 of all 77 successfully analysed samples (73%) were characterised as Prn2. These data correspond to data from other European countries where a dominance of the Prn2 variant was also observed [18,21]. Twelve percent of the isolated Austrian strains had the Prn1/7 isotype and in 16% of the strains the Prn3 variant was found (Table 2). We noted a small difference between these variants: whereas the Prn1/7 isotype was found in the whole sampling period, no more Prn3 samples were found after 2005.

**Table 2:** Genomic variations in *prn* variants of Austrian *B*. *pertussis* samples (n = 77) collected from 2002–2008. Frequencies are given as numbers and as percentage of successfully typed samples (in brackets).

City	Prn1/7^1^	Prn2	Prn3
Vienna (n = 25)	5 (20%)	18 (72%)	2 (8%)
Graz (n = 11)	1 (9%)	7 (64%)	3 (27%)
Linz (n = 41)	3 (7%)	31 (76%)	7 (17%)
Total (n = 77)	9 (12%)	56 (72%)	12 (16%)

^1^To distinguish the prn variants Prn1 and Prn7, the sequence of the second polymorphic region is needed as well.

### Little diversity found in SNPs in genes coding for virulence-associated factors

In order to include more polymorphisms in genes encoding virulence-associated factors, we addressed the SNPs discovered by comparing the Tohama I and L517 strains [20] in the *ptxA*, *ptxB*, *fimD*, *tcfA* and *bvgS* genes. All our samples (n = 110) were examined by ARMS-qPCR. Surprisingly, the genotype of all the successfully analysed samples was a mixed one of *bvgS*, *fimD*, *ptxA*, *ptxB* alleles as determined for strain L517 [20] and the tcf*A* allele as reported for Tohama I [19]. The results of the individual samples are shown in S1 Table.

From one reference isolate from Linz (P11), we amplified the region around the SNPs and performed classical Sanger sequencing. The obtained nucleotide sequences are available from the NCBI database with the accession numbers JF990849 (*ptxA*), HM185483 (*ptxB*), JF990848 (*fimD*), HM185484 (*tcfA*), and JF990847 (*bvgS*). All sequences confirmed the previous results of the ARMS-qPCR for the SNPs. In all cases except *ptxA*, no additional sequence variations were found: The *bvgS* sequence was 100% identical to strains 18323, CS and BP165, and displayed only one SNP, G489-A, compared to the Tohama I strain. The *fimD* sequence was 100% identical to strains 18323 and CS, with a single SNP, C138-T, compared to the strain Tohama I. The *ptxB* sequence was identical to strains BP165, 18323 and 3779, whereas strains CS, B1917, B1920, B1834, B1831, and Tohama I displayed one SNP A87-G. Finally the *tcfA* sequence has been sequenced more frequently, our sequence was identical to 46 strains including Tohama I and had SNPs with five strains. In contrast, the *ptxA* sequence displayed slightly more polymorphisms, the A490-G SNP was found in strains B1917, B1920, B1834 and B1831. In strain BP165 and strain 3779 there was another SNP from A192-G. In strain CS and Tohama I there were two SNPs A490-G and A192-G. Strain 18323 displayed three SNPs, T94-C, A192-G and A204-G.

### High prevalence of the *ptxP3* allele in the pertussis toxin promoter region

Finally, we examined our samples for the presence of the *ptxP3* allele, possessing the base “A”in the *ptxA* promoter region in position -65 relative to the transcriptional start site [22]. All other *ptxP* alleles (here called *ptxP1*-like) contain the base “G”in this position. Strains carrying the *ptxP1* allele such as the strain Tohama I express lower levels of Ptx than the *ptxP3* strains which have become more prevalent in recent years [23].

We used ARMS-qPCR to test for the *ptxP3* and *ptxP1*-like alleles in our samples (S1 Table). In total, we found the *ptxP3* allele in as many as 71 out of 76 amplified samples (93%). Only five of the amplified samples (7%) carried the *ptxP1* allele. Interestingly, four out of these five were of the Prn1/7 isotype. The *ptxP1* samples were from Linz (two samples from 2007) and Vienna (three samples from 2003).

## Discussion

In Austria, similar to many other countries with routine vaccination programs against pertussis, the disease is far from eradication, and the number of cases has been increasing since the year 1995. To explain this rising prevalence, one of the current hypotheses is that under vaccine pressure *B*. *pertussis* may adapt slowly via the selection of mutant strains. The aim of this study was to examine DNA polymorphisms in *B*. *pertussis* strains found in the Austrian population.

The first method applied (n = 34 samples analysed successfully) was MLVA typing [17] which addresses regions with DNA repeats. As observed before, this method revealed the highest level of polymorphisms. MLVA Type 27 dominated as seen in other countries during a similar sampling period [18]. MLVA Type 27 has seven repeats of the VNTR6 sequence, whereas Type 29 has nine repeats of this sequence [17]. The sequence VNTR6 is found in a pseudogene of a membrane protein, so the difference between the MLVA types does not appear to be related to a difference in an expressed protein. Nevertheless, the relative prevalence of MLVA Types 27 and 29 changed drastically during the past decades. In isolates from the United Kingdom, MLVA Type 29 had been found frequently towards the end of the last century, but had slowly disappeared thereafter [31]. This change was associated with decreasing prevalence of strains carrying the allele *ptxP1* in the pertussis toxin promoter region, and increasing prevalence of *ptxP3* strains expressing higher levels of Ptx [32]. In the light of these findings, it is not surprising that no more MLVA Type 29 was found in our Austrian specimen collected between 2002 and 2006.

In the genes coding for vaccine antigens, variable region-1 of the *prn* gene is the region with the highest sequence diversity [14] and this region was examined by PCR amplification followed by conventional Sanger sequencing (n = 77 successfully determined). In total, 13 variants of *prn* had been found [21], but in our study, only the variants Prn1/7 (n = 9), Prn2 (n = 56), and Prn3 (n = 12), and none of the variants Prn4–Prn6 and Prn8–Prn13 were found, with Prn2 strongly dominating (72%). As we had amplified and sequenced only the variable region-1, the variants Prn1 and Prn7 could not be distinguished as they differ only in one base position in the variable region-2.

The increase in the prevalence of Prn2 pertussis strains is associated with the re-emergence of this pathogen in many countries, from Argentina and France [33,34], the USA [35], the United Kingdom [36], the Netherlands [14], France [34], Poland [37], Finland and Sweden [38], to Russia [39]. Unexpectedly, Australia [40] and Japan [41] turned out to be an exception with the Prn1 variant dominating, but in Australia this situation changed drastically in the 2008–2010 epidemic when 86% of the samples were found to be Prn2 variants [42].

A further important recent change is the rising prevalence of the virulent *ptxP3* strains with a predicted higher expression of Ptx [23]. The vast majority (93%) of the tested Austrian samples, which had been collected between 2002 and 2008, carried the *ptxP3* allele. The remaining five *ptxP1*-like samples were from 2003 and 2007, their number is too small to conclude a decrease over time.

During the 2008–2010 epidemic in Australia [42], 84% of the samples displayed the *ptxP3* / Prn2 combination. Similarly, 77% of our Austrian samples were analysed as *ptxP3* / Prn2. Whereas in Australia, only 2% of the *ptxP3* samples were non-Prn2 variants [42], 16% of the Austrian samples were *ptxP3* / Prn3 and 1% *ptxP3* / Prn1. Taken together, our analysis showed that in Austria the *ptxP3* / Prn2 strains represent the majority as was found in many other countries around the world. Nevertheless, other variants were present at a significant percentage.

Taken together, our data agree with a number of findings from other countries. *B*. *pertussis*, a monomorphic pathogen [21] displays only limited variability on the level of SNPs, so addressing some of these failed to reveal any variability. A higher variability is always found in repetitive elements such as the pentapeptide repeats of Prn in the variable region-1 and the repeats addressed by the MLVA method. Although the rate of changes is not high, the changes need to be monitored carefully. The availability of whole genome sequences will facilitate the identification of additional polymorphic regions. Two examples for this approach are the analysis of six additional *B*. *pertussis* genomes including four *ptxP1* and two *ptxP3* strains [43], and the whole genome sequence analysis of as many as 343 strains by Illumina technology [44] which revealed a number of further interesting polymorphisms, that should be included in future studies. As the strains in the latter study covered a long period of 90 years and were from many countries, an overall picture of the strain evolution could be provided [44]. One of the results of the study was the absence of a geographical clustering of strains, apparently *B*. *pertussis* strains such as the newly evolved *ptxP3* lineage spread rather rapidly. Therefore it is not surprising that our Austrian results reflect many developments from other countries such as the high prevalence of the Prn2 isotype and *ptxP3* allele or the absence of MLVA Type 29 in samples from the recent years. Although in the global comparison of the 343 strains [44] the average density of SNPs was found to be one SNP per 770 bp, it was higher in the virulence-associated than in the housekeeping genes, demonstrating the potential of *B*. *pertussis* to slowly escape from vaccine pressure.

**Fig 1:**
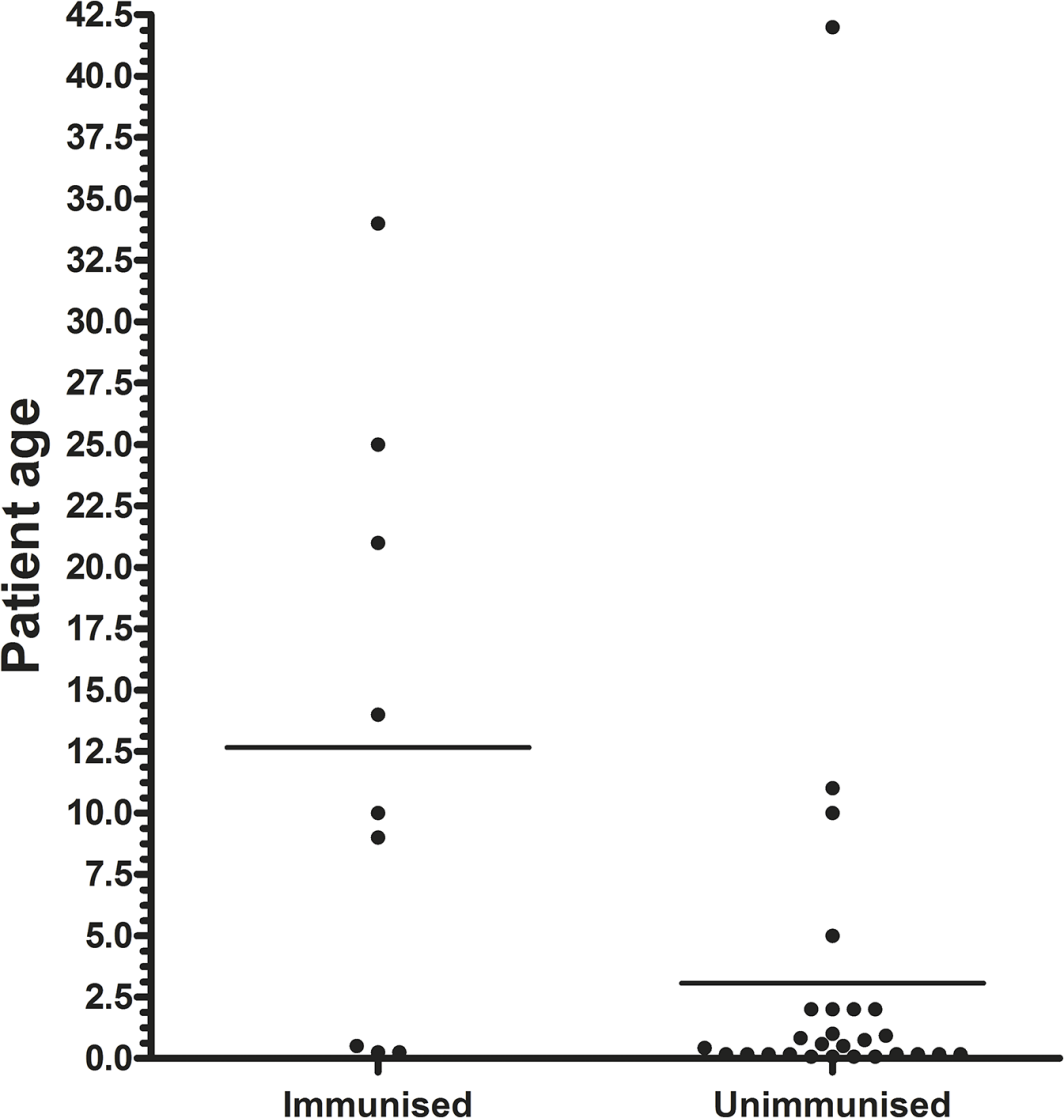
Age distribution of immunised (n = 9) and unimmunised (n = 27) pertussis patients. The immunisation status of the patients was unknown in many cases (see S1 Table).

Among the Austrian pertussis patients with known immunisation status (n = 46), 74% had not been vaccinated, and this remains the main risk factor for aquiring the disease. Within each group of the major genotypes, the MLVA isotype 27, the Prn2 isotype and the *ptxP3* isotype, 80% of the patients had not been immunised. Though not being the main focus of our study, we also compared the ages of the patients that had suffered from pertussis in spite of being immunised with those patients that had not been immunised (Fig 1). The median age of immunised patients was 10 years, and of unimmunised patients only 0.5 years, significantly lower (p<0.005). If the immunised individuals get the disease later than the unimmunised ones, it goes to show that the immunisation works, but there is also a waning immunity.

In order to limit the further spread of pertussis in a population with high vaccine coverage, the first remedy would therefore be a booster vaccination at school entry (6 years) to counteract the waning of antibody levels. Additionally, regular booster vaccination every 10 years during adulthood, followed by every 5 years after the age of 60 are strongly recommended by the Austrian Advisory Committee on Immunization [45], but this new strategy has not yet been broadly implemented.

## Materials and Methods

### Ethics statement

The samples collected from pertussis patients during an extended time period were taken as part of routine diagostics and not for the purpose of this study. We obtained aliquots and strictly anonymised patient information limited to age, and where available, whether the patients had received an immunisation including pertussis. For this type of surveillance, ethical evaluation or patient consent are not required.

### Study sites and collection of samples

Pertussis patients were diagnosed as suffering from cough for at least 14 days, in addition one of the following symptoms: paroxysmal cough, inspiratory whoop, or posttussive vomiting [46]. A total of 110 *B*. *pertussis* samples were collected from 2002 to 2008 in three Austrian cities (Vienna (n = 32), Linz (n = 63), and Graz (n = 15)). Only the first part of these samples collected from 2002 to 2006 (n = 77) was studied by MLVA typing. All 110 samples including 33 samples collected later were characterised in the other parts of this study. Bacterial DNA was isolated from nasopharyngeal swabs of *B*. *pertussis* infected patients using the QIAamp DNA Mini Kit according to the manufacturer’s protocol (Qiagen, Hilden, Germany).

### Multiple-Locus Variable-Number Tandem Repeat Analysis (MLVA)

Six VNTRs, (VNTR1, VNTR3a, VNTR3b, VNTR4, VNTR5 and VNTR6) were PCR-amplified by using forward primers 5´-labelled with 6-carboxyfluorescein and unlabelled reverse primers as reported [17] with bacterial DNA isolated from *B*. *pertussis* infected patients serving as template. The amplification products were analysed with an ABI Prism 3100 Genetic Analyzer (Life Technologies) at VBC Biotech, Vienna, Austria. The number of repeats found at the six VNTR loci translates into a distinct MLVA type (www.mlva.net/).

### Sanger sequencing of *prn* alleles

So far, 13 *prn* alleles have been identified with variation mainly limited to two regions [14]. For our study, region 1 was chosen for sequencing as most variations are found therein. Primers were designed as described [14] and are displayed in Table 3. The *prn* gene was PCR-amplified from bacterial DNA isolated from *B*. *pertussis* infected patients. A second PCR with the amplicon of the first PCR as the template was used to increase the sensitivity. The first PCR reaction (10 μl volume) consisted of 1× PCR Buffer (Qiagen) containing 1.5 mM MgCl_2_, 1× Q-Solution (Qiagen), 300 μM of each dNTP (Thermo Scientific), 0.5 μM of each primer (PrnAF and PrnAR [14]), 1 μl bacterial genomic DNA and 0.5 units HotStarTaq DNA Polymerase (Qiagen). The reaction was incubated at 95°C for 15 min, followed by 30 cycles of 94°C for 10 s, 50°C for 45 s and 72°C for 1 min, and final elongation at 72°C for 10 min. In the second PCR (30 μl volume), 1.5 μl of the 100-fold diluted amplicon of the first PCR were used as input. Cycling conditions were the same as for the first PCR, except for the number of cycles (45 cyles instead of 30) and the annealing temperature (62°C instead of 50°C). PCR cycling was performed in a Mastercycler gradient (Eppendorf, Hamburg, Germany).

**Table 3:** Primers and probes used in this study. **A,** Primers prnAF (top) and prnAR (bottom) [14] were used for *prn* sequencing. **B,** **C-1,** ARMS-qPCR primers for the determination of the *ptxA*, *ptxB*, *fimD*, *bvgS* and *tcfA* alleles **(B)** as well as for the discrimination between the *ptxP1*-like and *ptxP3* alleles **(C-1)**. For each gene, the forward primer is shown on top, then the reverse primer corresponding to the Tohama I sequence and the reverse primer corresponding to the alternative allele, with the base at the SNP site underlined, and finally the labelled hydrolysis probe. Lower case letters, artificial base replacement with both sequences at the SNP site; FAM, 6-carboxyfluorescein; TET, 4,5,6,7-tetrachlorofluorescein; BHQ1, Black Hole Quencher-1; Cy5, red sulfoindocyanine dye. **C-2,** oligonucleotides for the *fimD* non-discriminatory control assay.

Gene	5’ Nucleotide position^1^	Sequence	Length [bp]	T_m_ [°C]	Amplicon length [bp]
**A**: PCR primers for sequence analysis of *prn* alleles [14]					
*prn*	55187	GCCAATGTCACGGTCCAA	18	57	586
	55772	GCAAGGTGATCGACAGGG	18	56	
**B:** Oligonucleotides for typing the *ptxA*, *ptxB*, *fimD*, and *tcfA* alleles by ARMS-qPCR					
*ptxA*	160397	AACCCCTACACATCGCGAAGGT	22	63	73
	160469	CGCCTATCACCGGCcCC	17	63	
	160469	CGCCTATCACCGGCcCT	17	60	
	160445	Cy5-CCAATGTGCCGACGATCGACGC TACG-BHQ1	26	74	
*ptxB*	160878	CCATCGTAGAGCGCAAATATTG	22	58	148(+1)^2^
	160731	AACAGATTACCCAGCATGtCC	21	53	
	160730	GAACAGATTACCCAGCATGtCA	22	56	
	160765	FAM-CTGCGCGAACAAGACCCGTG CC-BHQ1	22	72	
*fimD*	250435	CGGCCGCAGTCCTATGG	17	60	65(+1)^2^
	250500	GGTCTCCTCCGTGGAGCaGA	20	61	
	250499	GTCTCCTCCGTGGAGCaGG	19	59	
	250453	FAM-CATCCGGGCATCGTGGTCGA CTTGC-BHQ1	25	74	
*tcfA*	221437	GGTTGCCCGGTATAGGAAAGGT	22	61	134
	221570	ATCCTTGCCCGCCCaAC	17	60	
	221570	ATCCTTGCCCGCCCaAT	17	60	
	221513	FAM-CATGGCGTCCGGAGCGG-BHQ1	17	66	
*bvgS*	232055	CGACTCGCTGGGCGAACT	18	61	92
	231964	CCTTGGCGTCGTGCAGaTT	19	61	
	231964	CCTTGGCGTCGTGCAGaTC	19	61	
	232025	FAM-CGGCGGCTGGATCGACATCA CCG-BHQ1	23	75	
**C:** Duplex qPCR for typing of *ptxP3* and *ptxP1*-like alleles					
1 –ARMS-qPCR assay					
*ptxP*	159628	CTACTGCAATCCAACACGGC	20	58	68(+1)^2^
*ptxP1*-like	159695	GACGGTGACCGGTtCCA	17	57	
*ptxP3*	159696	GGACGGTGACCGGTtCTA	18	55	
	159655	TET-CTCCTTCGGCGCAAAGTCG CG-BHQ1	21	70	
2 –Consensus (non-discriminatory) control assay					
*fimD*	250434	CCGGCCGCAGTCCTATG	17	60	113
	250546	CCTTCTGCATCGGCGAACT	19	60	
	250453	FAM-CATCCGGGCATCGTGGTCGA CTTGC-BHQ1	25	74	

^1^The nucleotide numbering refers to the numbering from the Tohama I genome [19]. Segment accession numbers: BX640414 (*prn*), BX640422 (*ptxP*, *ptxA*, *ptxB*), BX640416 (*fimD* and *bvgS*) and BX640414 (*tcfA*).

^2^The “colder”primer forming only two hydrogen bonds at the specific base has a one base extension at its 5´-end to compensate the reduced T_m_.

The amplicon was purified using the QIAquick PCR Purification Kit according to the manufacturer’s protocol. The amplification primers were used for Sanger sequencing of both DNA strands (GATC Biotech, Konstanz, Germany).

### Amplification refractory mutation system quantitative PCR (ARMS-qPCR) to detect alleles of *ptxA*, *ptxB*, *fimD*, *tcfA* and *bvgS* genes as well as the ptxP3 and ptxP1-like alleles

ARMS-qPCR [30], uses a SNP-specific primer having a specific base at the penultimate or last position of the 3´-end and mismatching one or two bases upstream of this specific nucleotide with the two (or three) sequences at the SNP site. This design strongly reduces unspecific primer extension resulting in a limit for SNP discrimination of down to 0.1% [47]. ARMS-qPCR was performed in the hydrolysis probe detection format. T_m_ values were calculated by means of the software Primer Express 2.0 (Life Technologies). The T_m_ of ARMS primers is given for the completely matching sequence (Table 3).

Singleplex qPCR for detecting the target SNPs in *ptxA*, *ptxB*, *fimD*, *tcfA* and *bvgS* genes was performed in a 15 μl reaction in 96-well plates using the StepOnePlus Real Time PCR System running under software version 2.0 (Life Technologies). The reaction contained 1 × PCR buffer B2 (Solis Biodyne, Tartu, Estonia), 4.5 mM MgCl_2_, 0.2 mM of each dNTP, 250 nM consensus primer, 150 nM ARMS primer, 150 nM hydrolysis probe, 1 U HOT FIREPol DNA Polymerase (5 U/μl, Solis Biodyne), and 8 ng DNA. In the case of the *tcfA* gene having a high GC content of 70%, the mastermix was supplemented with 1 × Solution S (Solis Biodyne). Initial denaturation for 15 min at 95°C was followed by 3 cycles at 95°C for 20 s and 66°C for 55 s, 3 cycles at 95°C for 20 s and 65°C for 55 s, 3 cycles at 95°C for 20 s and 64°C for 55 s, finally 60 cycles at 95°C for 20 s and 65°C for 55 s.

The 10 μl duplex qPCR for differentiating the *ptxP3* and *ptxP1*-like alleles contained 5 × HOT FIREPol Probe qPCR Mix Plus (no ROX) (Solis Biodyne), 250 nM of each primer, 100 nM of each hydrolysis probe, and 1.5 μl template DNA. Amplification performed on the ViiA 7 Real-Time PCR System (Life Technologies) was started by an initial denaturation for 10 min at 95°C, and followed by 50 cycles at 95°C for 15 s, 58°C for 25 s, and 60°C for 25 s.

### Statistical analysis

Genotypic diversity (GD) based on MLVA typing was calculated by the equation: GD = [n/(n-1)](1-∑x_i_^2^), where x_i_ is the frequency of the i^th^ DNA type and n is the number of strains [18,48,49].

The age distributions between immunised and non-immunised patients were compared by means of the two-tailed Wilcoxon–Mann–Whitney test.

## Supporting Information

S1 Table List of all samples and typing results (see separate file). (DOC)
