# Supplementary material for: Genetic Variation of Bordetella pertussis in Austria
Source: PLoS One. 2015 Jul 16;10(7):e0132623. doi: 10.1371/journal.pone.0132623 (PMC4504479; doi:10.1371/journal.pone.0132623)
Supplement: S1 Table — (DOC) [file pone.0132623.s001.doc]

Supplementary Table 1. Genotyping results of Austrian *Bordetella pertussis* isolates

| **No.** | **Sample name** | **Sample origin** | **Patient**  **age** | **Sampling year** | **Immun. status^1^** | **MLVA type** | ***prn* variant** | **Genotyping results^2^ for target SNPs in**  ***bvgS*** ***fimD*** ***ptxA*** ***ptxB*** ***tcfA*** ***ptxP3*** | | | | | | **SNP sites genotyped** |
| --- | --- | --- | --- | --- | --- | --- | --- | --- | --- | --- | --- | --- | --- | --- |
| 1 | P1 | Linz | 0.5 | 2004 | 0 | 27 | Prn2 | L | L | L | f.a. | f.a. | + | 4 |
| 2 | P2 | Linz | 12 | 2004 | 2 | 27 | Prn2 | L | L | L | L | f.a. | + | 5 |
| 3 | P3 | Linz | 2 | 2005 | 0 |  | f.a. | f.a. | f.a. | f.a. | f.a. | f.a. | f.a. | 0 |
| 4 | P4 | Linz | 1 | 2005 | 0 | 27 | Prn3 | L | L | L | f.a. | f.a. | + | 4 |
| 5 | P5 | Linz | 0.1 | 2005 | 0 | 27 | Prn2 | L | L | f.a. | L | f.a. | + | 4 |
| 6 | P6 | Linz | 21 | 2005 | 1 | 27 | Prn2 | L | L | L | f.a. | f.a. | + | 4 |
| 7 | P7 | Linz | 0.2 | 2005 | 0 | 27 | f.a. | f.a. | f.a. | L | f.a. | f.a. | f.a. | 1 |
| 8 | P8 | Linz | 0.5 | 2005 | 1 | 36 | Prn2 | L | L | L | f.a. | f.a. | + | 4 |
| 9 | P9 | Linz | 0.1 | 2005 | 0 | 38 | Prn2 | L | L | L | L | f.a. | + | 5 |
| 10 | P10 | Linz | 25 | 2005 | 1 | 38 | f.a. | L | f.a. | f.a. | f.a. | f.a. | f.a. | 1 |
| **11** | **P11** | **Linz** | 0.2 | 2005 | 0 | 38 | Prn2 | **L** | **L** | **L** | **L** | **T** | **+** | **6** |
| 12 | P12 | Linz | 0.6 | 2005 | 0 |  | f.a. | L | f.a. | f.a. | f.a. | f.a. | f.a. | 1 |
| 13 | P13 | Linz | 0.3 | 2005 | 0 | 38 | Prn2 | L | L | f.a. | L | f.a. | + | 3 |
| 14 | P14 | Linz | 0.8 | 2005 | 2 |  | Prn3 | L | L | f.a. | L | f.a. | + | 4 |
| 15 | P15 | Linz | 0.3 | 2005 | 2 | 27 | Prn3 | L | f.a. | f.a. | L | f.a. | + | 2 |
| 16 | P16 | Linz | 10 | 2005 | 0 |  | f.a. | L | f.a. | f.a. | f.a. | f.a. | f.a. | 1 |
| 17 | P17 | Linz | 1 | 2005 | 0 | 63 | Prn3 | f.a. | f.a. | f.a. | f.a. | f.a. | f.a. | 0 |
| 18 | P18 | Linz | 0.3 | 2005 | 1 | 27 | Prn2 | L | L | f.a. | L | f.a. | + | 4 |
| 19 | P19 | Linz | 0.3 | 2005 | 2 | 27 | Prn3 | L | L | L | L | f.a. | + | 5 |
| 20 | P20 | Linz | 42 | 2005 | 0 |  | f.a. | L | f.a. | f.a. | f.a. | f.a. | + | 2 |
| 21 | P21 | Linz | 0.2 | 2005 | 0 |  | Prn3 | L | L | L | L | f.a. | + | 5 |
| 22 | P22 | Linz | 0.4 | 2005 | 0 | 128 | Prn2 | L | L | L | L | f.a. | + | 5 |
| 23 | P23 | Linz | 30 | 2005 | 2 |  | f.a. | L | L | f.a. | L | f.a. | f.a. | 3 |
| **24** | **P24** | **Linz** | ? | **2005** | 0 | 27 | Prn3 | **L** | **L** | **L** | **L** | **T** | **+** | **6** |
| 25 | P25 | Linz | 0.2 | 2005 | 0 | 27 | Prn2 | L | L | f.a. | L | f.a. | + | 4 |
| 26 | P26 | Linz | 37 | 2005 | 2 | 27 | Prn2 | L | L | f.a. | L | f.a. | + | 4 |
| 27 | P27 | Linz | 2 | 2005 | 0 | 63 | f.a. | L | f.a. | f.a. | f.a. | f.a. | f.a. | 1 |
| 28 | P28 | Linz | 0.2 | 2005 | 0 | 27 | f.a. | f.a. | f.a. | f.a. | f.a. | f.a. | f.a. | 0 |
| 29 | P29 | Linz | 2 | 2005 | 0 | 27 | Prn2 | L | L | f.a. | L | f.a. | + | 4 |
| 30 | P30 | Linz | 47 | 2005 | 2 | 27 | Prn2 | L | L | f.a. | L | f.a. | + | 4 |
| **No.** | **Sample name** | **Sample origin** | **Patient**  **age** | **Sampling year** | **Immun. status** | **MLVA type** | ***prn* variant** | **Genotyping results^1^ for target SNPs in**  ***bvgS*** ***fimD*** ***ptxA*** ***ptxB*** ***tcfA*** ***ptxP3*** | | | | | | **SNP sites genotyped** |
| 31 | P31 | Linz | 0.1 | 2005 | 2 | 27 | Prn2 | L | f.a. | L | L | f.a. | + | 4 |
| 32 | P32 | Linz | 2 | 2005 | 2 |  | f.a. | L | f.a. | f.a. | f.a. | f.a. | f.a. | 1 |
| 33 | P33 | Linz | 0.4 | 2005 | 2 | 27 | Prn2 | L | L | L | L^3^ | f.a. | + | 5 |
| 34 | P34 | Linz | 0.8 | 2005 | 0 | 129 | Prn2 | L | f.a. | f.a. | f.a. | f.a. | f.a. | 1 |
| 35 | P35 | Linz | 1 | 2005 | 2 | 27 | Prn2 | L | L | f.a. | L | f.a. | + | 4 |
| 36 | P36 | Linz | 2 | 2006 | 0 |  | f.a. | L | f.a. | f.a. | f.a. | f.a. | f.a. | 1 |
| 37 | P37 | Linz | 10 | 2006 | 1 |  | f.a. | L | f.a. | f.a. | f.a. | f.a. | f.a. | 1 |
| 38 | P38 | Linz | 0.5 | 2006 | 2 | 27 | Prn2 | L | f.a. | f.a. | L | f.a. | + | 2 |
| 39 | P39 | Linz | 2 | 2006 | 2 |  | Prn2 | L | f.a. | f.a. | L | f.a. | + | 3 |
| 40 | P40 | Linz | 34 | 2006 | 1 |  | f.a. | L | f.a. | f.a. | f.a. | f.a. | f.a. | 1 |
| 41 | P41 | Linz | 0.2 | 2006 | 0 |  | Prn2 | L | f.a. | f.a. | L | f.a. | + | 3 |
| 42 | P42 | Linz | 0.2 | 2006 | 0 |  | Prn2 | L | L | f.a. | f.a. | f.a. | + | 3 |
| 43 | P43 | Linz | 0.3 | 2007 | 2 |  | Prn2 | L | L | L | L | f.a. | + | 5 |
| 44 | P44 | Linz | 0.3 | 2007 | 2 |  | Prn2 | L | f.a. | L | f.a. | f.a. | + | 3 |
| 45 | P45 | Linz | 0.3 | 2007 | 2 |  | Prn2 | L | L | f.a. | both | f.a. | + | 4 |
| 46 | P46 | Linz | 0.2 | 2007 | 2 |  | Prn2 | L | L | L | L | f.a. | + | 5 |
| 47 | P47 | Linz | 0.4 | 2007 | 2 |  | Prn1/7 | L | L | f.a. | L | f.a. | - | 4 |
| 48 | P48 | Linz | 32 | 2007 | 2 |  | f.a. | L | L | f.a. | L | f.a. | - | 4 |
| 49 | P49 | Linz | ? | 2007 | 2 |  | Prn1/7 | L | f.a. | f.a. | f.a. | f.a. | f.a. | 1 |
| 50 | P50 | Linz | ? | 2007 | 2 |  | f.a. | L | f.a. | f.a. | f.a. | f.a. | f.a. | 1 |
| 51 | P51 | Linz | ? | 2007 | 2 |  | f.a. | L | f.a. | f.a. | f.a. | f.a. | f.a. | 1 |
| 52 | P52 | Linz | 0.1 | 2007 | 0 |  | Prn2 | L | L | f.a. | L | f.a. | + | 4 |
| 53 | P53 | Linz | ? | 2007 | 0 |  | f.a. | L | f.a. | f.a. | f.a. | f.a. | f.a. | 1 |
| 54 | P54 | Linz | 5 | 2007 | 0 |  | Prn2 | L | L | f.a. | L | f.a. | + | 4 |
| 55 | P55 | Linz | 0.8 | 2007 | 0 |  | Prn2 | L | f.a. | f.a. | L | f.a. | + | 3 |
| 56 | P56 | Linz | ? | 2007 | 2 |  | f.a. | L | f.a. | f.a. | f.a. | f.a. | f.a. | 1 |
| 57 | P57 | Linz | 8 | 2007 | 2 |  | Prn1/7 | f.a. | L | f.a. | L | f.a. | + | 3 |
| 58 | P58 | Linz | ? | 2007 | 0 |  | f.a. | L | f.a. | f.a. | L | f.a. | + | 3 |
| 59 | P59 | Linz | 8 | 2007 | 2 |  | Prn2 | L | L | f.a. | L | f.a. | + | 4 |
| 60 | P60 | Linz | 0.3 | 2008 | 1 |  | Prn2 | L | L | L | L | f.a. | + | 5 |
| 61 | P61 | Linz | ? | 2008 | 0 |  | f.a. | L | f.a. | f.a. | f.a. | f.a. | f.a. | 1 |
| 62 | P62 | Linz | ? | 2008 | 1 |  | f.a. | L | f.a. | L | f.a. | f.a. | + | 3 |
| 63 | P63 | Linz | 1 | 2008 | 2 |  | f.a. | L | f.a. | f.a. | f.a. | f.a. | + | 2 |
| 64 | G01 | Graz | 0.2 | 2002 | 2 |  | Prn3 | L | L | L | L | f.a. | + | 5 |
| 65 | G02 | Graz | 0.2 | 2002 | 2 | 36 | Prn2 | L | f.a. | L | L | f.a. | + | 4 |
| **No.** | **Sample name** | **Sample origin** | **Patient**  **age** | **Sampling year** | **Immun. status** | **MLVA type** | ***prn* variant** | **Genotyping results^1^ for target SNPs in**  ***bvgS*** ***fimD*** ***ptxA*** ***ptxB*** ***tcfA*** ***ptxP3*** | | | | | | **SNP sites genotyped** |
| 66 | G03 | Graz | 16 | 2002 | 2 | 27 | Prn3 | L | f.a. | f.a. | L | f.a. | + | 3 |
| 67 | G04 | Graz | 1 | 2002 | 2 |  | Prn2 | f.a. | f.a. | f.a. | f.a. | f.a. | f.a. | 0 |
| 68 | G05 | Graz | 13 | 2002 | 2 |  | f.a. | L | f.a. | f.a. | f.a. | f.a. | f.a. | 1 |
| 69 | G06 | Graz | 0.3 | 2002 | 2 |  | Prn2 | L | L | L | f.a. | f.a. | + | 4 |
| 70 | G07 | Graz | 5 | 2002 | 2 |  | Prn2 | L | L | L | L | f.a. | + | 5 |
| 71 | G08 | Graz | 0.4 | 2004 | 2 |  | Prn2 | L | f.a. | f.a. | L | f.a. | + | 3 |
| 72 | G09 | Graz | 0.3 | 2002 | 2 | 27 | Prn3 | L | f.a. | L | L | f.a. | + | 4 |
| 73 | G10 | Graz | 0.3 | 2002 | 2 | 27 | Prn2 | L | L | f.a. | L | f.a. | + | 4 |
| 74 | G11 | Graz | 10 | 2003 | 2 |  | f.a. | f.a. | f.a. | f.a. | L | f.a. | f.a. | 1 |
| 75 | G12 | Graz | 0.3 | 2002 | 2 |  | f.a. | f.a. | f.a. | f.a. | f.a. | f.a. | f.a. | 0 |
| 76 | G13 | Graz | 0.4 | 2005 | 2 | 18 | Prn2 | L | L | L | f.a. | f.a. | + | 4 |
| 77 | G14 | Graz | 0.3 | 2005 | 2 |  | Prn1/7 | f.a. | f.a. | f.a. | L | f.a. | f.a. | 1 |
| 78 | G15 | Graz | ? | ? | 2 | 27 | f.a. | f.a. | f.a. | f.a. | f.a. | f.a. | f.a. | 0 |
| **79** | **S01** | **Vienna** | **0.1** | **2003** | **2** |  | Prn2 | **L** | **L** | **L** | **L** | **T** | **+** | **6** |
| 80 | S02 | Vienna | 0.1 | 2003 | 2 |  | Prn2 | L | L | L | L | f.a. | + | 5 |
| 81 | S03 | Vienna | 12 | 2003 | 2 |  | Prn3 | L | L | L | L | f.a. | + | 5 |
| 82 | S04 | Vienna | 0.2 | 2003 | 2 |  | Prn2 | L | L | L | L | f.a. | + | 5 |
| 83 | S05 | Vienna | 0.1 | 2003 | 2 |  | Prn2 | L | L | L | L | f.a. | + | 5 |
| 84 | S06 | Vienna | 0.2 | 2003 | 2 |  | f.a. | L | L | L | L | f.a. | + | 5 |
| **85** | **S07** | **Vienna** | **40** | **2003** | **2** |  | Prn1/7 | **L** | **L** | **L** | **L** | **T^3^** | **-** | **6** |
| **86** | **S08** | **Vienna** | **1** | **2003** | **2** |  | Prn1/7 | **L** | **L** | **L** | **L** | **T** | **-** | **6** |
| 87 | S09 | Vienna | 4 | 2003 | 2 |  | Prn1/7 | L | L | L | L | f.a. | - | 5 |
| 88 | S10 | Vienna | 0.4 | 2003 | 2 |  | Prn2 | L | L | L | L | f.a. | + | 5 |
| **89** | **S11** | **Vienna** | **0.3** | **2003** | **2** |  | Prn2 | **L** | **L** | **L** | **L** | **T** | **+** | **6** |
| 90 | S12 | Vienna | 0.2 | 2003 | 2 |  | Prn3 | L | L | L | L | f.a. | + | 5 |
| **91** | **S13** | **Vienna** | **0.1** | **2003** | **2** |  | Prn2 | **L** | **L** | **L** | **L** | **T** | **+** | **6** |
| 92 | S14 | Vienna | 0.2 | 2003 | 2 |  | Prn2 | L | L | L | L | f.a. | + | 5 |
| **93** | **S15** | Vienna | **0.1** | **2003** | **2** |  | Prn2 | **L** | **L** | **L** | **L** | **T** | **+** | **6** |
| 94 | S16 | Vienna | 0.2 | 2003 | 2 |  | Prn2 | L | L | L | L | f.a. | + | 5 |
| 95 | S17 | Vienna | 0.2 | 2003 | 2 |  | Prn2 | L | L | L | L | f.a. | + | 5 |
| **96** | **S18** | **Vienna** | **0.1** | **2003** | **2** |  | Prn2 | **L** | **L** | **L** | **L^3^** | **T** | **+** | **6** |
| 97 | S19 | Vienna | 0.2 | 2004 | 2 |  | Prn2 | L | L | L | L | f.a. | + | 5 |
| **98** | **S20** | **Vienna** | **0.7** | **2004** | **2** |  | Prn2 | **L** | **L** | **L** | **L** | **T^3^** | **+** | **6** |
| 99 | S21 | Vienna | 5 | 2004 | 2 |  | Prn2 | f.a. | f.a. | f.a. | L | f.a. | + | 2 |
| 100 | S22 | Vienna | 0.1 | 2004 | 2 |  | Prn2 | L | L | L | f.a. | T | + | 5 |
| **No.** | **Sample name** | **Sample origin** | **Patient**  **age** | **Sampling year** | **Immun. status** | **MLVA type** | ***prn* variant** | **Genotyping results^1^ for target SNPs in**  ***bvgS*** ***fimD*** ***ptxA*** ***ptxB*** ***tcfA*** ***ptxP3*** | | | | | | **SNP sites genotyped** |
| 101 | W01 | Vienna | 9 | 2007 | 1 |  | Prn1/7 | f.a. | f.a. | f.a. | f.a. | f.a. | f.a. | 0 |
| 102 | W02 | Vienna | 0.9 | 2007 | 0 |  | Prn2 | L | f.a. | f.a. | f.a. | f.a. | f.a. | 1 |
| 103 | W03 | Vienna | ? | 2007 | 0 |  | f.a. | L | f.a. | f.a. | L | f.a. | f.a. | 2 |
| 104 | W04 | Vienna | 1 | 2007 | 0 |  | Prn2 | L | L | L | L | f.a. | + | 5 |
| 105 | W05 | Vienna | ? | 2007 | 0 |  | f.a. | f.a. | f.a. | f.a. | L | f.a. | f.a. | 1 |
| 106 | A01 | Vienna | ? | 2007 | 1 |  | f.a. | L | f.a. | f.a. | f.a. | f.a. | f.a. | 1 |
| 107 | A02 | Vienna | ? | 2007 | 2 |  | f.a. | f.a. | f.a. | f.a. | L | f.a. | f.a. | 1 |
| 108 | A03 | Vienna | 14 | 2008 | 1 |  | Prn1/7 | L | f.a. | f.a. | L | f.a. | f.a. | 2 |
| 109 | A04 | Vienna | ? | 2008 | 1 |  | f.a. | L | f.a. | f.a. | f.a. | f.a. | f.a. | 1 |
| **110** | **A05** | **Vienna** | ? | **2008** | **0** |  | f.a. | **L** | **L** | **L** | **L** | **T** | **+** | **6** |

^1^Immun.status: immunization status, 0, non-immunized; 1, immunized; 2, status not known.

^2^SNP variants genotyped by ARMS-qPCR were assigned either to the *B. pertussis* strains L517 (L) or Tohama I (T) or to the *ptxP3* (+) or non-*ptxP3* alleles (-).

^3^confirmed by Sanger sequencing.

f.a., failed amplification.

Samples in bold were genotyped at all six loci.
